# Potential for micronuclear turnover through autophagy secretion pathway

**DOI:** 10.17912/micropub.biology.001545

**Published:** 2025-03-11

**Authors:** Natsu Asami, Sarasa Yano, Fuminori Tsuruta

**Affiliations:** 1 Master's Program in Biology, Degree Programs in Life and Earth Sciences, Graduate School of Science and Technology, University of Tsukuba, 1-1-1 Tennodai, Tsukuba, Ibaraki 305-8577, Japan; 2 Graduate School of Life and Environmental Sciences, University of Tsukuba, 1-1-1 Tennodai, Tsukuba, Ibaraki 305-8577, Japan; 3 Master's and Doctoral Program in Biology, Institute of Life and Environmental Sciences, University of Tsukuba, 1-1-1 Tennodai, Tsukuba, Ibaraki 305-8577, Japan; 4 Master's and Doctoral Program in Neuroscience, Graduate School of Comprehensive Human Sciences, University of Tsukuba, 1-1-1 Tennodai, Tsukuba, Ibaraki 305-8577, Japan; 5 Doctoral Program in Human Biology, Graduate School of Comprehensive Human Sciences, University of Tsukuba, 1-1-1 Tennodai, Tsukuba, Ibaraki 305-8577, Japan; 6 Doctoral Program in Humanics, School of Integrative and Global Majors, University of Tsukuba, 1-1-1 Tennodai, Tsukuba, Ibaraki 305-8577, Japan; 7 Center for Quantum and Information Life Sciences, University of Tsukuba, 1-1-1 Tennodai, Tsukuba, Ibaraki 305-8577, Japan

## Abstract

Micronuclei (MN) serve as well-established markers of genomic instability. MN arise from various stresses, such as segregation errors and mechanical stress, and are subsequently eliminated by the autophagy pathway. It has been suggested that MN are traditionally considered markers of cancer cells, often without recognized functional significance. Meanwhile, we recently discovered that MN act as mediators in regulating microglial characteristics. Neurons produce MN in response to migrating stress during the developmental stage and release them to the extracellular space, subsequently transferring them to microglia. In this study, we report the potential mechanisms underlying MN release through the autophagic secretion pathway. Our data show a possibility by which damaged MN are recognized autophagy regulatory factors, resulting in the propagation of MN to microglia.

**Figure 1. Autophagy pathway is associated with micronuclei secretion.**
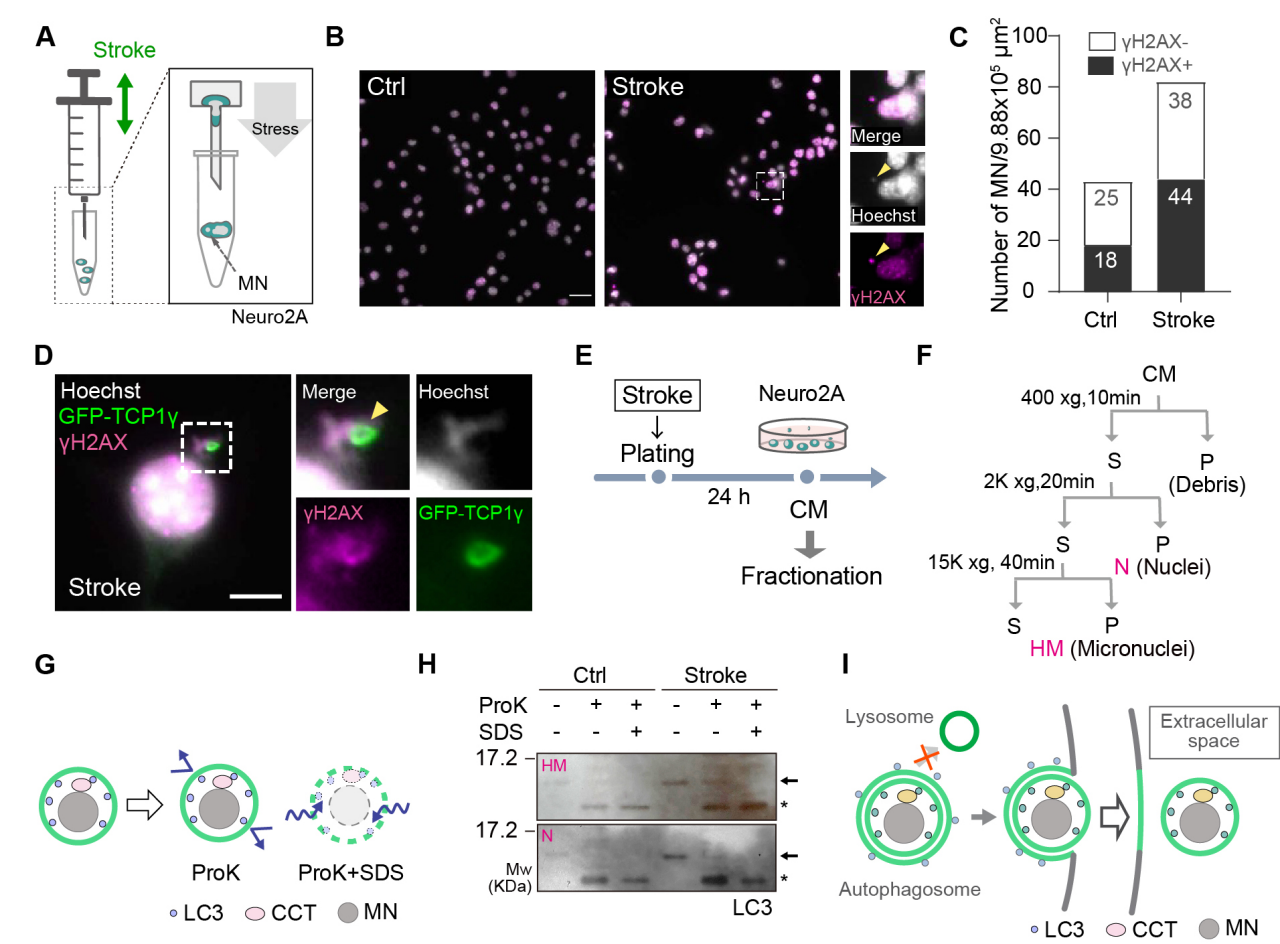
**(A)** Schematic illustration of MN formation *in vitro* via mechanical stress. Neuro2A cells were stimulated by syringe pumping. MN: micronuclei. (B) Neuro2A cells were plated for 24 hours after 10 strokes and were then stained with Hoechst and anti-γH2AX antibody. Scale bar: 20 μm. **(C)** The number of γH2AX ^+^ and γH2AX ^-^ MN were counted. The mechanical stress increased the number of γH2AX ^+^ MN. n=10 field (1 field/9.88x10 ^4^ μm). Data were reproduced in three independent experiments. **(D)** Neuro2A cells were transfected with GFP-TCP1γ plasmid and were then stimulated by pumping after harvesting cells. After 24 hours of incubation, cells were stained with anti-GFP and γH2AX antibodies. Ratio of γH2AX ^+^ GFP-TCP1γ ^+^ Hoechst ^+^ MN to γH2AX ^+^ Hoechst ^+^ MN is approximately 60%. Scale bar: 5 μm. **(E)** Schematic diagram for fractionation of MN. CM: conditioned medium. **(F)** The flow of fractionation. Heavy membrane fractions were collected as a fraction containing MN. CM: conditioned medium, S: supernatant, P: pellet, N: nuclear fraction, HM: heavy membrane fraction. **(G)** Schematic illustration for proteinase K (ProK) protection assay. In the case of MN surrounded by a membrane, MN are protected from degradation by ProK treatment, and LC3 proteins within the vesicles are detected. When the membrane is removed by sodium dodecyl sulfate (SDS) treatment, the proteins in the vesicles are degraded and cannot be detected. **(H)** Immunoblotting of the extracellular MNs after ProK protection assay. The arrows indicate LC3, and asterisks indicate non-specific bands. **(I)** The working hypothesis of MN secretion is dependent on the autophagy pathway. The image is modified from our paper (Yano et al., 2025).

## Description

MN are extranuclear structures produced by mitotic chromosomal imbalances and DNA damage (Krupina et al., 2021; Zych and Hatch, 2024). They also arise from mechanical stress as the cell passes through a narrow region (Denais et al., 2016; Raab et al., 2016). Subsequently, MN undergo degradation through the autophagy pathway (Rello-Varona et al., 2012). Recent studies have suggested that MN formation is implicated in chromothripsis and metastasis of cancer cells (Bakhoum et al., 2018; Crasta et al., 2012). These observations imply that MN, produced in response to various stresses, serve as novel mediators that underlie regulating cellular properties. Normally, MN are encased by a nuclear envelope. However, when this membrane is compromised, the chromatin in the MN leaks into the cytoplasm, which is recognized by the DNA sensor cGAS. This reaction promotes cGAMP production and STING activation, subsequently triggers an innate immune response (Dou et al., 2017; Harding et al., 2017; Mackenzie et al., 2017). It has been recently shown that the disruption of the MN envelope is associated with reactive oxygen species (ROS) derived from mitochondria. The ECSRT-III complex plays a crucial role in repairing damaged nuclear envelopes. Mitochondrial ROS oxidizes the cysteine residues of CHMP7, a scaffold protein in the ECSRT-III complex, thereby inhibiting the repair of the nuclear envelope (Di Bona et al., 2024). Additionally, mitochondrial ROS promotes the aggregation of p62, a protein essential for the selective proteolysis involved in autophagy, on the MN membrane. Notably, p62 selectively degrades CHMP7 through the autophagy pathway rather than degrading the MN (Martin et al., 2024).

Recently, we have reported propagation of neuronal MN regulates microglial characteristics during the developmental brain (Yano et al., 2025). We found that migrating neurons receive mechanical stress from the surrounding environment, leading to the generation of MN during development. These MN are released at the superficial area of the cerebral cortex and subsequently recognized and incorporated by microglia. Importantly, the incorporation of MN alters microglial morphology and gene expression patterns. Thus, we propose that MN serve as novel mediators regulating microglial characteristics (Yano et al., 2025). However, the mechanisms underlying the release of MN into extracellular space remain an open question. Here, we propose a potential mechanism showing that MN damaged by mechanical stress can be secreted into the extracellular space via the autophagy pathway.

We first investigated the link between mechanical stress-induced MN and DNA damage. Previously, we found that neurons after pumping with a small syringe produce MN (Yano et al., 2025) ( Figure 1A ). Using *in vitro* MN formation assay system, we stained Neuro-2A cells together with the DNA damage marker, phospho-Histone H2A.X at Ser139 (γH2AX) (Rogakou et al., 1998). Consistent with our previous study, the number of MN was increased after pumping of syringe in Neuro-2A cells. Importantly, γH2AX-positive MN were upregulated ( Figure 1B and 1C), suggesting that MN formation produced by mechanical stress is associated with DNA damage. Previously, T-complex protein ring complex (TRiC) has been reported to serve as an adaptor between LC3 and aggregated proteins, inducing autophagy degradation (Ma et al., 2022). We also found that TRiC family proteins localized in the MN (Yano et al., 2025). Thus, we analyzed the link between TCP1γ, which is a component of TRiC family protein and γH2AX. We transfected GFP-TCP1γ plasmid into Neuro-2A cells, conducted *in vitro* MN formation assay. Consequently, GFP-TCP1γ was colocalized with γH2AX signals in the MN ( Figure 1D ). These observation prompt us to speculate that γH2AX is a potential target of TCP family protein for autophagosome formation though we could not detect direct binding by co-immunoprecipitation assay. It has been thought that autophagic pathway is also involved in not only a degradation but also a secretion (Ponpuak et al., 2015). Since we observed that extracellular MN are covered by membrane using a transmission electron microscope (Yano et al., 2025), we presumed that MN secretion occurs utilizing the autophagy secretion pathway. To examine this idea, we performed the proteinase K protection assay ( Figure 1E to 1G) (Tan et al., 2022). We first confirmed whether mechanical stress induces MN secretion by detecting LC3-II, which localizes MN fractions (Rello-Varona et al., 2012; Yano et al., 2025). The amount of LC3 in the MN fractions were increased in response to mechanical stress. Furthermore, LC3 was not significantly degraded by proteinase K treatment and residual LC3 was detected. In contrast, SDS treatment eliminated LC3 ( Figure 1H ). These results suggest that extracellular MN are encased in a membrane.

In summary, the autophagy secretion pathway represents a potential mechanism for releasing MN into the extracellular space. We propose that γH2AX-positive sites serve as potential targeting sites for TRiC family proteins, which act as adaptors between LC3 and the MN. The recognition of MN by TRiC family proteins may trigger the autophagy secretion pathway ( Figure 1I ). Taken together, our data suggest that MN are released via intracellular vesicular formation.

## Methods

**Antibodies, materials, and plasmids**

For immunofluorescence, anti-γH2AX [JBW30, Cat# 05-636 (1:1000), Millipore (Burlington, MA, US)] and anti-GFP [ab13970 (1:500), abcam (Cambridge Biomedical Campus, Cambridge, UK)] antibodies were used as the primary antibody. For immunoblotting, anti-LC3 antibody was purchased from MBL [PM036 (1:500), MBL (Tokyo, Japan)]. Proteinase K was purchased from Fujifilm WAKO (Osaka, Japan). The TCP1γ cDNA was amplified from HCT116 cells cDNA library and subcloned into the EcoRI site of pCS4-EGFP. The TCP1γ primers used were as follows: forward, 5'-AACAATTGGCCACCATGATGGGCCATCGTCCAGTGCTCG-3', and reverse, 5'-AACAATTGTCACTCCTGGCCAGCATCAGGAGC-3';

**Cell culture and transfection**

Neuro-2A cells were purchased from JCRB Cell Bank. Neuro-2a cells were cultured in Eagle's minimum essential medium (EMEM) (FUJIFILM Wako) containing 10% fetal bovine serum (FBS) and P/S (FUJIFILM Wako) in a 37°C incubator with 5% CO _2_ . Neuro-2A cells were transfected with GFP-TCP1γ plasmid using polyethyleneimine MAX (PEI Max) (Polyscience, Warrington, PA, US). The amount of the plasmids and PEI Max were optimized in proportion to the relative surface area and the number of cells (Taketomi et al., 2022). Briefly, Neuro-2a cells were at 1.0 x 10 ^6^ cells/well on 10 cm dishes and incubated at 5% CO _2_ and 37 °C for one day. The plasmids (4.0 μg) were mixed with 200 μl of Opti-MEM (Thermo Fisher) and 1.0 µg/µl of PEI Max (16.0 μl) was mixed with 200 μl of Opti-MEM in another tube. Both solutions were combined and incubated for 20 min at room temperature, followed by adding these mixtures to cells.

**Immunocytochemistry** Neuro-2A cells were plated on 10 cm dishes for one day and were then transfected with GFP-TCP1γ plasmid. After one day incubation, Neuro2A cells were stimulated with 10 strokes of syringe pumping using a needle [No.32 (0.26 mm x 12 mm), HS-2739B, Dentronics], re-plated at 2.5 × 10 ^4^ cells on glass coverslips (15 mm diameter, Matsunami Glass) in 12 well plates, and were then subjected to immunochemical analysis.

Neuro-2A cells were fixed with 4% paraformaldehyde in phosphate-buffered saline (PBS) for 10 min at room temperature (RT) and were blocked in in 0.25% Triton X-100 in blocking solution (5% BSA in PBS) for 30 min at RT. Then, cells were incubated with anti-GFP and anti-γH2AX antibodies diluted in blocking solution for overnight at 4°C. After washing with PBS, the cells were incubated with goat anti-chicken IgY (H+L) secondary antibody, Alexa Fluor 488 [Cat # A-11039 (1:1000), Thermo Fisher Scientific] diluted in blocking solution for 30 minutes at room temperature. The nuclei were stained with 10 μg/ml Hoechst 33342 (SIGMA). The coverslips were mounted onto slide glasses using FLUOROSHIELD Mounting Medium (ImmunoBioScience, Davis, CA, US). Fluorescent images were obtained using the fluorescence microscope (Keyence, BIOREVO BZ-9000) equipped with 40x (PlanApo 40x/0.95) and 100x (PlanApo VC 100x/1.4 Oil) objective and a charge-coupled device camera (Keyence). The images were acquired using BZ-II software (Keyence).

**Quantification of MN**

The imaging data were analyzed by the MATLAB-based program CAMDi (Yano et al., 2021). The rate of overlap area of the nucleus is defined as 0.5. The imported data are conducted binarization and are determined the threshold of each color. After making the binary images, the merged areas are extracted according to the threshold, followed by quantification of the number and area. Thus, the extra nuclei within a major diameter of 3.0 μm were defined as MN.

**Proteinase K protection assay**

Neuro-2A cells were stimulated with syringe pumping (10 strokes) and were then plated at 1.0 x 10 ^6^ cells/well on 10 cm dishes for one day. The collected conditioned media were collected using trypsin and were subjected to cellular fractionation assay. The collected media were subjected to a centrifugation step of 400 x g for 10 min at 4℃ to remove cells. Next, the supernatant was centrifuged at 2,000 x g for 20 min at 4℃ to remove nuclei after rupturing by apoptosis. The supernatant was centrifuged at 15,000 x g for 40 min at 4℃. The precipitated samples from media were extracted with 100 μl of buffer (10 mM Tris-HCl [pH7.5], 150 mM NaCl, 1 mM EDTA) and were subjected to proteinase K protection assay (Tan et al., 2022). The samples containing 100 μg/ml of proteinase K (Fujifilm WAKO) were incubated at 37℃ for 30 min. Trichloroacetic acid solution (final concentration; 10%) (Fujifilm WAKO) were added into the samples and incubated for 30 min on ice. After removing supernatant, the pellets were mixed with 200 μl of ice-cold acetone, centrifuged at 14,000 rpm for 4 min at 4℃ twice. collected precipitated samples, and subjected to an immunoblot analysis.

**Immunoblot analysis**

The samples of proteinase K protection assay were conducted SDS-polyacrylamide gel electrophoresis. After running the gels, each sample was transferred to a polyvinylidene difluoride membrane (Pall). The membranes were blocked in 5% skim milk for 60 min at room temperature and were then incubated overnight at 4°C with the anti-LC3 antibody (MBL) as primary antibodies. After washing with PBS, the membranes were incubated for 1 hour with the Peroxidase AffiniPure Rabbit Anti-Mouse IgG + IgM (H+L) (Jackson ImmunoResearch, 315-035-048, 1:20000). The signals on the membrane were detected by Chemi-Lumi One Super (Nacalai tesque)

**Image processing**

Image processing were conducted using Adobe Creative Could CC (Photoshop 22.1.0 and Illustrator 25.0.1) (https://www.adobe.com/), FIJI Image J 2.1.0/1.53c (https://imagej.nih.gov/ij/).
